# Structural Instability of Human Serum Albumin during Microparticles Synthesis

**DOI:** 10.1021/acsabm.5c01228

**Published:** 2025-10-23

**Authors:** Elisa Fardelli, Giovanna De Simone, Radostina Georgieva, Yu Xiong, Michael Di Gioacchino, Simone Sotgiu, Alessandro Nucara, Angelo Tavella, Leonetta Baldassarre, Armida Sodo, Agnese Ricci, Tecla Gasperi, Paolo Ascenzi, Hans Bäumler, Alessandra di Masi, Giovanni Capellini

**Affiliations:** † Department of Sciences, University Roma Tre, 00146 Rome, Italy; ‡ Institute of Transfusion Medicine, Charité—Universitätsmedizin Berlin, 10117 Berlin, Germany; § Department of Medical Physics, Biophysics and Radiology, Faculty of Medicine, Trakia University, 6000 Stara Zagora, Bulgaria; ∥ Department of Physics, 9311 Sapienza University of Rome, 00185 Rome, Italy; ⊥ Laboratory of Nanomaterials for Environment and Health (NAMES), Biostructures and Biosystems National Institute (INBB), 00136 Rome, Italy; # Faculty of Pharmacy, Payap University, 50210 Chiang Mai, Thailand; ¶ IHP—Leibniz Institute for High Performance Microelectronics, 15236 Frankfurt (Oder), Germany

**Keywords:** human serum albumin, microparticles, heme, AFM, Raman spectroscopy

## Abstract

Human serum albumin, the most abundant plasma protein, plays vital roles in maintaining oncotic pressure, modulates drugs pharmacokinetics and pharmacodynamics, and features antioxidant and enzyme-like properties. This protein also exhibits high-affinity binding to a wide range of endogenous and exogenous molecules. Combined with its excellent solubility, biocompatibility, biodegradability, low toxicity, and nonimmunogenicity, these properties make human serum albumin an attractive platform for biomedical applications, particularly in drug delivery. This study reports a detailed physicochemical characterization of human serum albumin microparticles obtained via the coprecipitation-cross-linking-dissolution method. The resulting submicron particles are peanut-shaped, exhibit uniform morphology, and show robust mechanical properties, including high stiffness and colloidal stability. However, we observed that the microparticle agglomeration process induces significant structural changes in the protein. Notably, Raman and FTIR spectroscopies highlighted a partial switch from α-helices to β-sheets secondary structure, which leads to diminished HSA binding capability as here demonstrated in the case of hemin. This trade-off between mechanical integrity and biological activity poses challenges for applications requiring native protein interactions, such as the HSA-dependent efficient drug binding and controlled release. Conversely, the reduced binding capability of HSA molecules localized on the surface of MPs implies that these HSA-MPs cannot bind to plasma ligands (e.g., drugs, heme, bacteria toxins). This prevents unwanted ligands from being transported to cellular targets, ensuring that only those located within the HSA-MP are transported. Our findings open new avenues for engineering microsystems that could couple the structural resilience of these microparticles with restored functional surfaces.

## Graphical abstract

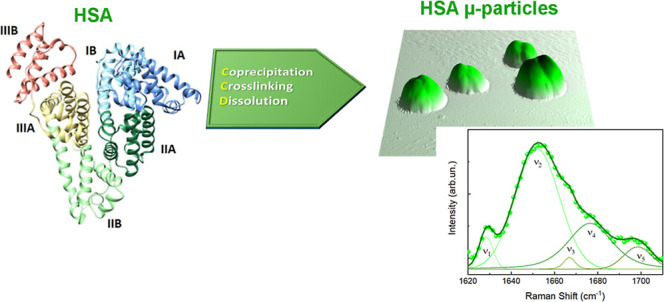

## 1 Introduction

Human serum albumin (HSA) is the most abundant plasma protein (∼60% of total plasma proteins) that regulates the oncotic pressure and the fluid distribution among the body compartments, affects pharmacokinetics of different drugs, and displays antioxidant and (pseudo)­enzymatic properties.^1–4^ HSA is a globular monomeric protein composed of 585 amino acids with a predominance of α-helices in the secondary structure. The molecule is organized in three domains encompassing amino acids 1–195 (domain I), 196–383 (domain II), and 384–585 (domain III) (Figure 1). Each domain is composed of two subdomains (i.e., A and B) connected by a long loop; subdomain A is composed of 6 α-helices and subdomain B of 4 α-helices, respectively.^5–8^ This structure confers extraordinary binding capacities to HSA. Indeed, HSA represents the main carrier of fatty acids (FAs) as well as endogenous and exogenous ligands (e.g., heme, metal ions, hormones, nucleic acids, drugs) at multiple binding sites known as FA1-FA9.^1,9–11^

**Figure 1:**
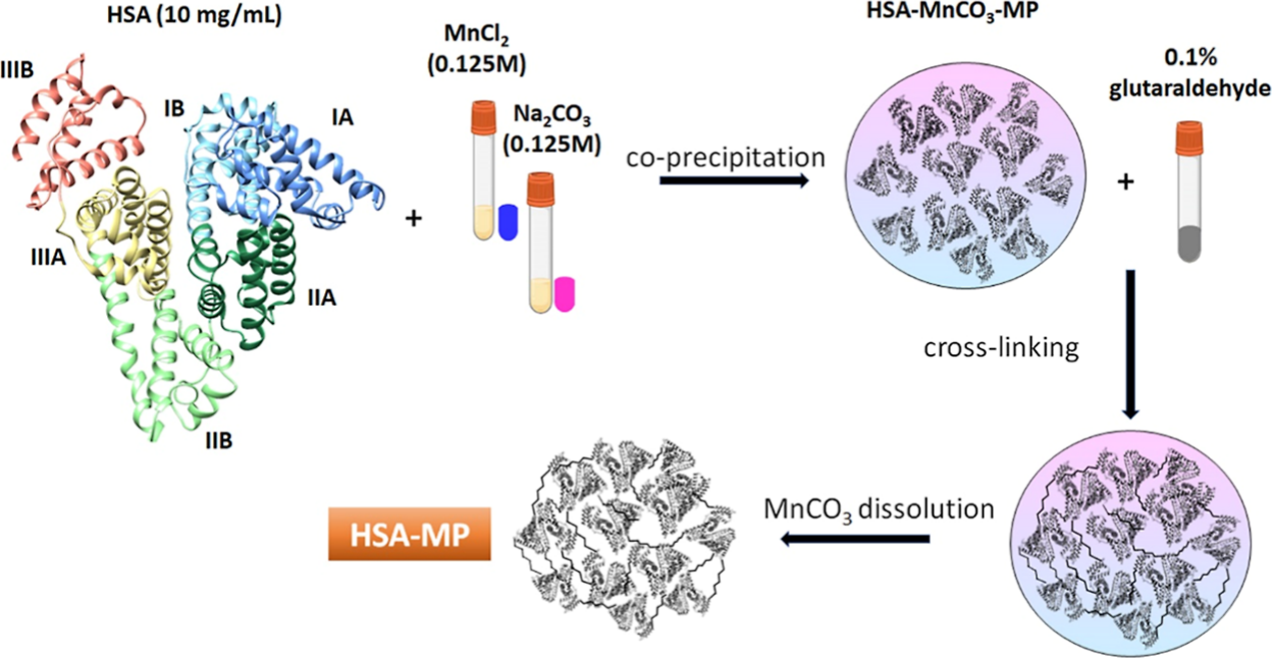
CCD protocol for the formation of HSA-MPs.

Since its initial clinical use in the 1940s as a blood substitute,^12^ albumin has evolved into a versatile tool in medicine, serving as carrier protein for therapeutic agents,^13^ as a diagnostic marker for conditions such as diabetes and kidney disease,^14^ and as a delivery platform for microparticle (MP) and nanoparticle (NP) formulations of chemotherapeutic drugs.^15^ HSA-MPs, HSA-NPs, nanoconjugates, and polyplexes possess unique properties, including small size, variable shape, and different binding sites for specific ligands (e.g., antibodies, peptides, and therapeutic nucleic acids). Their external shell is designed to protect the ligands from enzymatic degradation and nonspecific adsorption, thereby enhancing their half-life and transport. Additionally, HSA particles are nontoxic and nonimmunogenic.^11,15–17^ However, the fabrication of HSA-MPs or -NPs could potentially alter the protein structure and, consequently, its binding properties.^18–20^

In this work, we investigate the protein stability during the HSA-MP synthesis obtained by the coprecipitation-cross-linking-dissolution (CCD) technique.^21–24^ This approach, commonly used to fabricate biopolymer particles, enables the entrapment of proteins with high effectiveness by using appropriate inorganic compounds to induce precipitation. The peanut-shaped particles obtained by CCD exhibit a rather uniform submicron size distribution and mechanical stability. These MPs can be loaded with hydrophobic drugs such as doxorubicin^24^ or riboflavin^25^ after precipitation or simultaneously during coprecipitation. It is important to remember that the presence of HSA on the surface of MPs ensures their cellular internalization and, consequently, that of the ligands they contain. This is because HSA can recognize different receptors expressed on various cell types, including SPARC receptors that are overexpressed in cancer cells.^11^ Here, we observed that the MP synthesis process induces significant structural changes in HSA, with an increase in β-sheet content at the expense of α-helix content. This results in the HSA molecules exposed on the surface of the MPs having diminished binding capability with the prototypical HSA ligand, hemin, whose binding to HSA can easily be monitored using spectroscopic methods.^9,26,27^ Consequently, the HSA-MPs outer surface cannot bind to plasma ligands (e.g., drugs, metals, heme, bacterial toxins), thus preventing them from transporting unwanted ligands to cellular targets. To restore the binding capacity of the albumin present on the surface, it may be necessary to coat the mechanically stable HSA-MPs with a new layer of proteins.

## 2 Materials and Methods

### 2.1 Reagents

HSA (A3782, Merck KGaA, Darmstadt, Germany) was diluted in Milli-Q water to obtain the desired experimental concentrations. HSA concentration was confirmed spectrophotometrically at 280 nm using the ε_280_ nm value of 35.7 mM^–1^ cm^–1^. Hemin (i.e., oxidized form of heme; H9039, Merck KGaA) was dissolved in 100 mM NaOH to reach a final concentration of 50 mM. Hemin concentration was determined spectrophotometrically at 412 nm using the ε_412_ nm value of 192 mM^–1^ cm^–1^ of the pyridine–hemin adduct.^28,29^ Manganese chloride (MnCl_2_) tetrahydrate, sodium carbonate (Na_2_CO_3_), ethylenediaminetetraacetic acid disodium salt (EDTA), glutaraldehyde (GA) and sodium borohydride (NaBH_4_) were purchased from Sigma-Aldrich. Ampuwa (water and injectable) were purchased from Fresenius Kabi Deutschland GmbH.

### 2.2 HSA-MP Synthesis

HSA-MPs were fabricated as previously described.^30^ Briefly, equal volumes of 0.25 M of Na_2_CO_3_ and 0.25 M of MnCl_2_ containing 10 mg/mL HSA (Grifols Movaco S.A., Barcelona, Spain) were mixed rapidly for 30 s under vigorous stirring using a mechanical stirrer at room temperature. Followed by addition of 20% HSA solution to achieve a final HSA concentration of 7.5 mg/mL. After further 5 min incubation under stirring the suspension was centrifuged and the particles were washed twice (3000*g* for 3 min). The particles were suspended in GA solution (0.1% final concentration) and incubated at room temperature for 1 h, followed by centrifugation and quenching with 25 mM NaBH_4_. The MnCO_3_ templates were dissolved by adding EDTA solution (0.5 M, pH 7.4) at room temperature for 30 min. The resulting particles were centrifuged, washed three times (10,000*g* for 10 min) and finally suspended in Ampuwa (Figure 1).

### 2.3 Preparation of HSA:Hemin and HSA-MP:Hemin Complexes

HSA:hemin and HSA-MP:hemin complexes were prepared by incubating 15 μM hemin with either 15 μM HSA (HSA:hemin molar ratio of 1:1)^29^ or 0.5% suspension of HSA-MP. Incubation was carried out for 2 h at 4 °C under gentle shaking. To confirm the formation of HSA:hemin and HSA-MP:hemin complexes, UV–vis absorption spectra were carried out at room temperature (RT) in the λ = [380, 500] nm spectral range using a LAMBDA 365+ spectrophotometer (PerkinElmer, Waltham, Massachusetts, USA) using a quartz cuvette with 1 cm optical path length. Due to the strong light scattering of HSA-MPs, the sample HSA-MP:hemin was diluted 1:5 before UV–vis spectra acquisition. The absorption spectrum of 0.1% HSA-MPs was subtracted to the UV–vis spectrum of HSA-MP:hemin. Similarly, the absorption spectrum of 33 μM NaOH was subtracted to the UV–vis spectrum of both hemin and HSA:hemin samples.

### 2.4 Atomic Force Microscopy (AFM)

To investigate the MP morphology, we employed a Dimension ICON AFM (Bruker, Santa Barbara, CA) operating in ScanAsyst mode and equipped with a ScanAsyst-Air Bruker silicon probe (nominal elastic constant of 0.4 N/m and nominal tip radius of 5 nm). The mechanical properties were measured by operating the same tool in quantitative nanomechanical mapping (QNM) mode using OTESPA probes (nominal elastic constant of 26 N/m). The AFM/QNM measurements were conducted immediately after dropping 2.5 μL of 0.2% HSA-MP solution in Milli-Q water on a clean Si(001) wafer surface. As demonstrated by repeating AFM acquisition on the same area, the MPs did not move during the measurements.

### 2.5 Raman Spectroscopy

Raman measurements were performed at RT in backscattering geometry with an InVia Renishaw μ-Raman spectrometer coupled with a Leica DM2700 M confocal microscope (Renishaw, New Mills, UK). The λ = 532 nm Raman laser pump was focused by a Leica 50× LWD objective in a ∼1.0 μm^2^ spot, with a power density at the sample of 3 × 10^5^ Wcm^–2^. The scattered Raman light was dispersed by a diffraction grating featuring 1800 grooves/mm, enabling a spectral resolution of ∼1 cm^–1^, and then collected by a charged coupled device (1024 × 256 pixels) Peltier-cooled at −70 °C. This set up was employed to analyze a water solution of 200 μM HSA and a 0.5% suspension of precipitated HSA. This suspension was obtained by rapidly mixing equal volumes of 0.125 M of MnCl_2_ and Na_2_CO_3_ and 0.5% of HSA for 30 s under vigorous stirring using a mechanical stirrer.^24^

Prior to the measurements, the spectrometer was calibrated by using the Raman shift of the Si­(LO) mode (520.5 cm^–1^) acquired on the same Si(001) substrates used for the MPs deposition.

Raman spectra on 20% suspension of HSA-MP were instead acquired in backscattering geometry using a Bruker MultiRAM Fourier transform (FT)-Raman spectrometer equipped with a Nd:YAG pump laser emitting at λ = 1064 nm and with a liquid-nitrogen-cooled, single-element Ge detector. A 632.8 nm He–Ne reference laser beam was coaligned with the Raman signal through the interferometer, serving as an internal wavelength reference and providing the spectral calibration. Spectra were recorded over the 70–3600 cm^–1^ range.

### 2.6 Fourier Transform Infrared (FTIR) Spectroscopy

Samples made of a 20% suspension of HSA-MPs and of a 1 mM solution of HSA were analyzed by FTIR spectroscopy. Both samples were resuspended in heavy water (D_2_O) (Merck KGaA, Massachusetts, United States). The HSA-MP were initially suspended in water, then the solution underwent three consecutive cycles of freeze-drying followed by rehydration with D_2_O to achieve total hydrogen to deuterium exchange. Instead, HSA powder was diluted in D_2_O and two freeze-drying and rehydration cycles were necessary for the HSA solution. Spectra were acquired using a commercial FTIR interferometer (Bruker IFS 66v/S). The sample compartment was equipped with a custom-built transmission accessory comprising a steel cell sealed with CaF_2_ windows. The optical path was set to 75 μm using a Mylar spacer positioned between the windows. All spectra were recorded at a spectral resolution of 4 cm^–1^ by coadding 128 interferograms. Transmittance spectra were obtained by dividing the spectra of the HSA-MPs and HSA samples by that of pure D_2_O, used as the reference.

### 2.7 Data Analysis

Raw AFM images were processed and analyzed with the open source Gwyddion Software following procedures that were previously described.^31,32^ The processed images were analyzed with a Python routine for data merge. Raman and FTIR spectra were analyzed and graphed using IGOR 6.37 (WaveMetrics Inc., USA). UV raw data visualization was conducted using GraphPad Prism version 8.0 for Windows. Origin 9.0 (OriginLab Corporation) was employed for visualization of all AFM, DLS, and UV data.

## 3 Results and Discussion

### 3.1 Morpho-Mechanical Characterization

In Figure 2A is reported a 3.5 × 3.5 μm^2^ three-dimensional (3D) rendering of an AFM image of four typical HSA-MPs. The MPs feature a peanut-like shape, characterized by two fused spherical-like lobes aligned along one symmetry axis passing through their centers (*Y*-axis, red). For a quantitative description of their morphology, we have measured individual particle sizes, i.e., the length (l) along the *Y*-axis (red line) the width (*w*), along the orthogonal *X*-axis (blue) at the fusion of the two lobes. Although these quantities vary within the HSA-MP ensemble, with l typically ranging from 450 to 850 nm and w ranging from 250 to 650 nm, the peanut-like shape of the HSA-MP is preserved, as exemplary shown in Figure 2B. The average projected shape ratio (PSR), defined as the length-to-width ratio (l/*w*), was measured to be PSR = 1.5 ± 0.2, averaging over 20 individual MPs in different sample regions.

**Figure 2:**
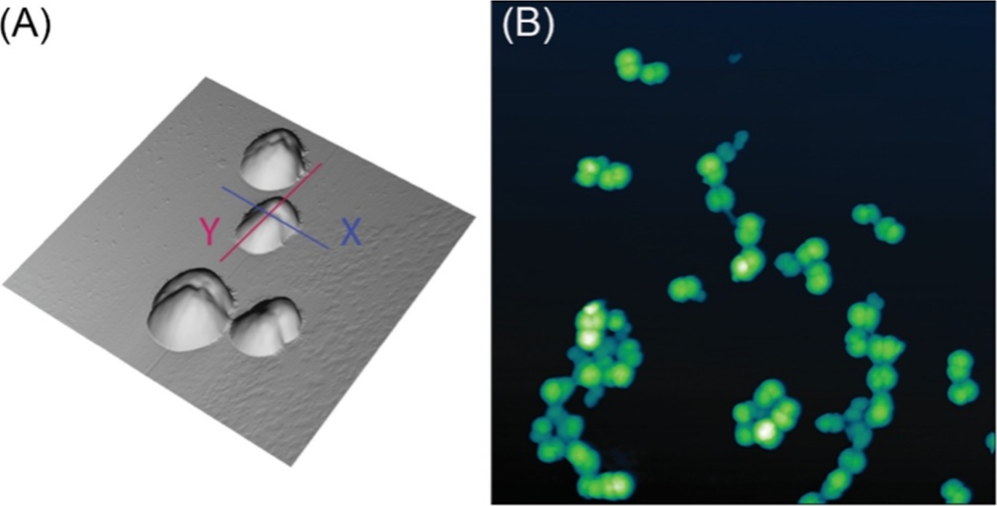
Morphology of HSA-MP. (A) 3.5 × 3.5 μm^2^ 3D rendering and (B) 10 × 10 μm^2^ AFM image of typical HSA-MPs.

To evaluate the average particle volume of the HSA-MP with statistical significance, while preserving size resolution, we adopted the following procedure. First, we calculated the volume ratio (VR), i.e., the ratio between the cube of the maximum height (*Z*_max_^3^) and the particle volume (*V*) of each individual HSA-MP (eq 1)­

1 $$VR=\frac{{Z_{\max}}^{3}}{V}$$

Indeed, the VR value can be used to better describe the MPs morphology. If we assume that the MP peanut-like shape visible in Figure 2 can be described by two spherical lobes, with *Z*_max_ being their equal diameter, we should expect a VR value ranging from VR = 3/π ∼ 0.95 (when the two lobes are tangent to each other) to VR = 6/π ∼ 1.91 (in the case the two spheres have merged in a single one having the same diameter).

To evaluate the VR average value, we measured *Z*_max_ and *V* on approximately 20 individual HSA-MPs found on a set of high lateral resolution 4 × 4 μm^2^ AFM images (see Figure 2A). The data show a linear behavior for the volume increase with *Z*_max_^3^, with a regression coefficient value of *R* ^2^ ≈ 0.97 (Figure 3A). This results in a VR = 0.26 ± 0.01, which is preserved independently of the size of the MPs of the investigated ensemble.

**Figure 3:**
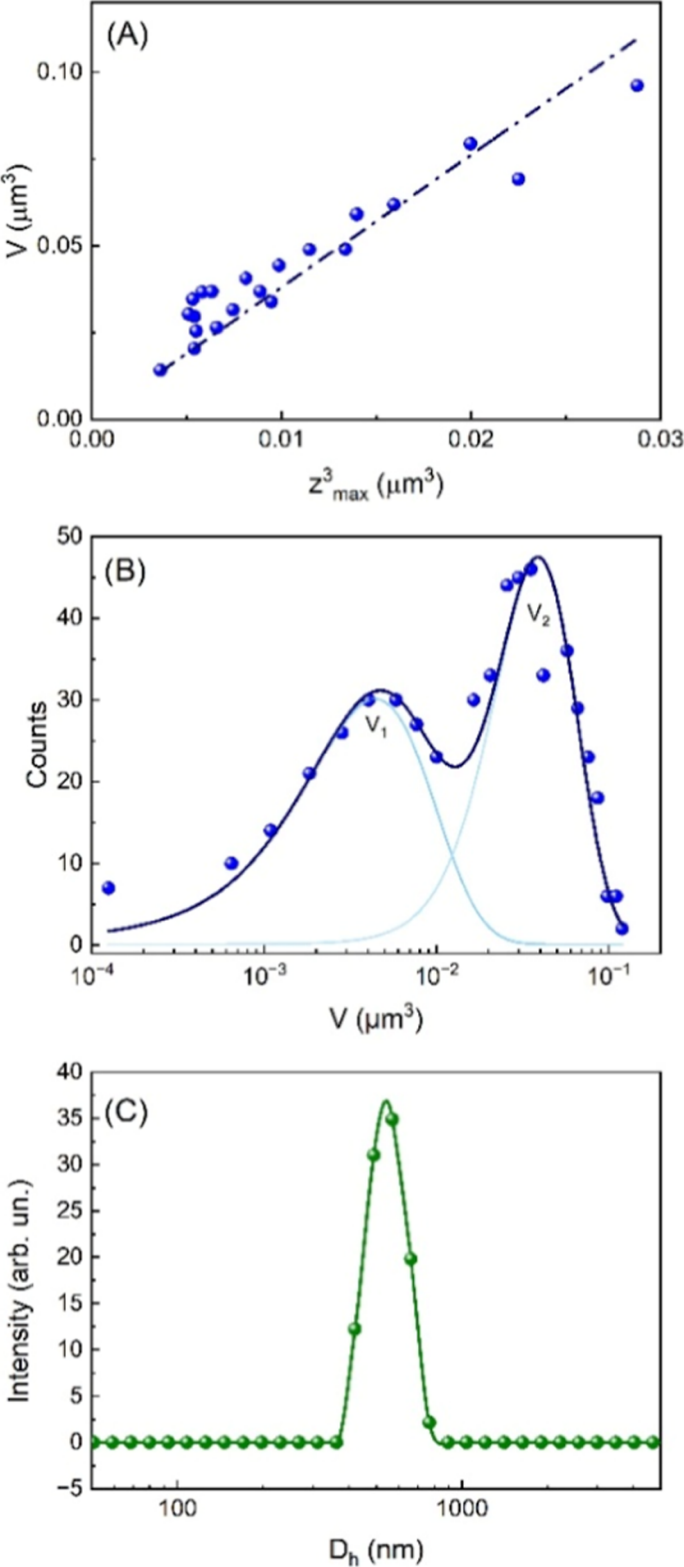
Quantitative analysis of the HSA-MP morphology. (A) Volumes of individual HSA-MP vs their relative *Z*_max_^3^ values. The dotted line represents the linear best fit. (B) Volume distribution obtained on 650 HSA-MPs (dots), deconvoluted with two Gaussian distributions. (C) Distribution of the DLS intensity of HSA-MP in solution.

This value is much lower of the ideal case of “hard-sphere peanutlike shape” above-discussed and indicates a more “flattened peanut-like” morphology.

As a second step toward the assessment of the volume statistical distribution, we acquired the MPs height *Z*_max_ on each of the ∼650 HSA-MPs found in ten large-field 30 × 30 μm^2^ AFM images. Although large-field AFM images are characterized by relatively poor lateral resolution (∼30 nm), their measurement resolution along the vertical *z*-direction, affecting the *Z*_max_ measurement, stays relatively high (we estimated it to be 0.1 nm), since it is not linked to the pixel size nor to the tip convolution affecting the lateral resolution. Since we have proven that VR was constant across the investigated population, *Z*_max_ can be then used to accurately calculate the MPs volume through eq 1. The resulting distribution, displayed in Figure 3B, show a bimodal distribution of the volume that can be approximated by two Gaussian distribution (adjusted *R* ^2^ ≈ 0.85) peaked at volumes of *V*_1_ = 4.7 × 10^–3^ μm^3^ and *V*_2_ = 3.8 × 10^–2^ μm^3^, respectively. The bimodal distribution reflects the two distinct populations (“small” and “large”) of HSA-MP that can be observed in Figure 2B.

As described in the Section 2, the HSA-MP solution was dropped on a substrate, dried, and immediately after investigated by AFM. To evaluate their size also in solution, thus mimicking their behavior in a biologically relevant environment, we have determined the hydrodynamic diameter distribution by DLS. We obtained an average value *D*_h_ = 540 ± 150 nm with a polydispersity index PDI = 0.45, indicative of a moderately broad size distribution, compatible with the AFM observations. We notice that both steric forces of HSA-MP and the measured negative zeta potential (−26.24 ± 1.25 mV) contribute to MP colloidal stability within the aqueous suspension.

By using the standard spherical approximation V_DLS_ = (*D*_h_)^3^·π/6, we obtain an average microparticle volume V_DLS_ ≈ 8 × 10^–2^ μm^3^. This value is roughly 20× and 2× larger than *V*_1_ and *V*_2_, respectively. We believe that this discrepancy can be explained by two different factors. On the one hand, the DLS measurements were carried out with the HSA-MPs in solution (i.e., MPs were hydrated), while the AFM measurements were carried out in a drying phase, leading to a loss of volume due to drying of HSA-MPs.^24^ On the other hand, the observed nonspherical, peanut-shaped morphology of the investigated HSA-MPs makes quantitative measurements of their dimensions difficult using the DLS technique. Specifically, although DLS can probe a large population of these anisotropic particles, it is limited in its ability to accurately resolve the hydrodynamic diameter and, consequently, infer the corresponding volume. This is because the technique approximates the MP as sphere, and the observed diameter represents a population-weighted average.^33^ This simplification, combined with the presence of a size distribution exhibiting a greater relative abundance of larger particles (Figure 3B), can result in a potential overestimation of the average hydrodynamic diameter (*D*_h_) and thus the apparent volume in DLS analysis. However, this technique is extremely useful for running comparative tests of particles from different batches and to verify the stability of the synthesis method.

Next, we investigated the mechanical properties of the HSA-MP using the QNM technique. The QNM enables the determination of stiffness, adhesion, and dissipation from force–distance curves obtained during interaction between an AFM tip and a sample surface. The stiffness reflects the material resistance to deformation and is related to the slope of the force–distance curve during contact, with higher stiffness indicating a harder or more rigid material. Adhesion refers to the attractive force exerted between the tip and the sample during the probe retraction cycle and indicates how “sticky” a material is. Dissipation quantifies the energy lost during each tip–sample interaction cycle and provides insights into material damping properties. Together, these parameters, along with the morphological maps, allow nanoscale morpho-mechanical characterization of the HSA-MPs. We acquired 20 QNM images (10 × 10 μm^2^), capturing the mechanical properties of approximately 600 HSA-MPs. From each image, we extracted the maps of stiffness (Figure 4A), dissipation (Figure 4B), and adhesion (Figure 4C). For each mechanical characteristic we extracted the mean value of each HSA-MP and computed their distributions. In detail, the three mechanical property distributions exhibit log–normal profiles (Figure 4), all with adjusted *R* ^2^ > 0.9. These results indicate that, regardless of morphological variations, the HSA-MPs feature uniform mechanical characteristics with the following values: stiffness ∼ 2.5 GPa, dissipation ∼ 6 keV, and adhesion ∼ 15 nN. Thus, the CCD-synthesized HSA-MPs are significantly stiffer and more adhesive compared to values reported in the literature for other nanoparticle systems used in pharmacological applications, such as hydrogels and HSA-TiO nanoparticles.^35–37^ These characteristics suggest that the HSA-MP could serve as a stable platform for biomedical applications. Notably, we point out here that their relatively large size may favor prolonged circulation in the bloodstream.^34–37^

**Figure 4:**
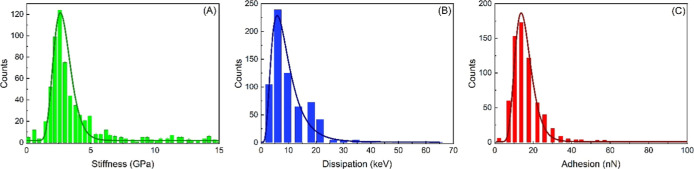
Distribution of the mechanical properties of HSA-MPs during dehydration. Distribution of (A) stiffness, (B) dissipation, and (C) adhesion of HSA-MPs. Experimental data (dots) were fitted with a log–normal curve (solid lines).

### 3.2 Chemical-Functional Characterization

After having assessed the morpho-mechanical properties of the HSA MPs, we now investigate the potential chemical modifications that HSA molecules could undergo during the HSA-MPs CCD synthesis process, possibly altering their capability to bind prototypical ligands.

To this end, Raman and FTIR spectroscopies were employed as tools to identify potential differences in the secondary structure of HSA and HSA-MPs. Indeed, these spectroscopies probe the roto-vibrational modes associated with molecular bonds of the sample, offering insight into its chemical composition and structural conformation. Figure 5 shows the Raman spectra of HSA (A) and HSA-MPs (B) in the region of the so-called Amide I band (1600–1700 cm^–1^). The spectra can be deconvolved into 5 different contributions, namely ν_1,_ ν_2_, ν_3,_ ν_4_, and ν_5_. Each of these “fingerprints” falls within a well-defined spectral range and is associated with a specific secondary structure of the protein, as described in Table 1.

**Figure 5:**
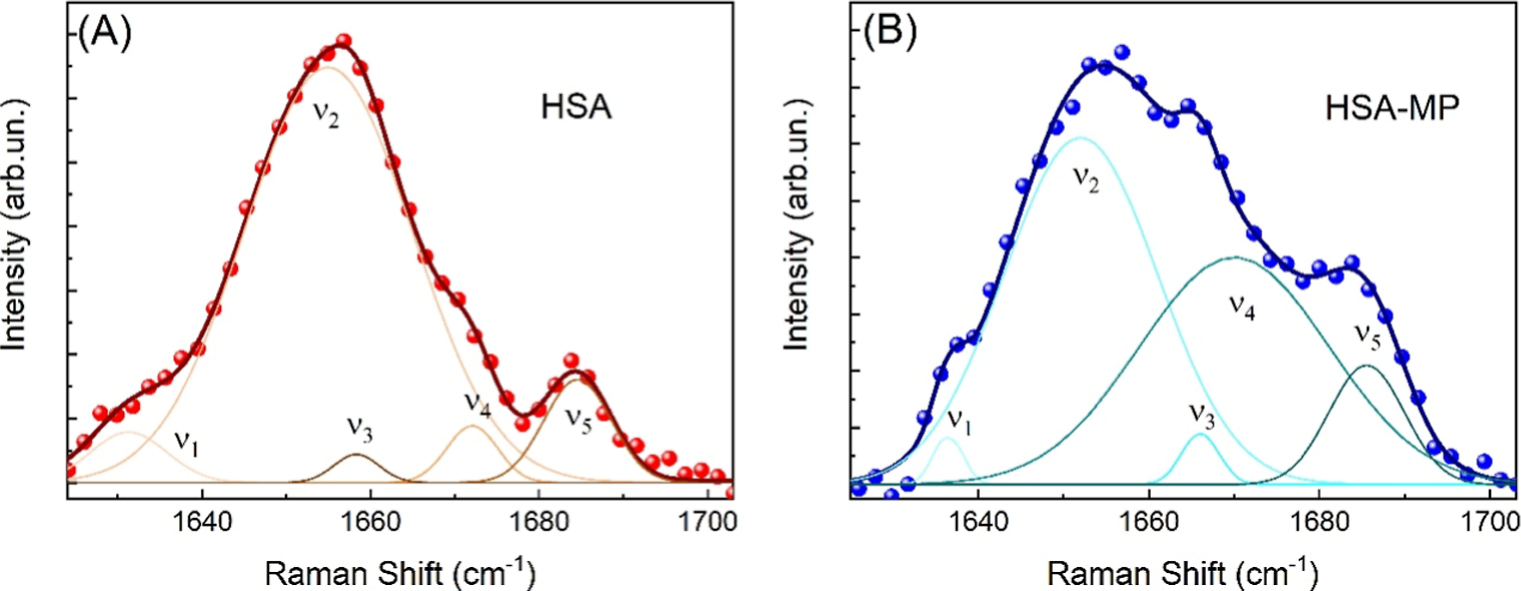
Comparison of Raman spectra of (A) HSA, and (B) HSA-MPs in the range of Amide I band, related to the protein secondary structure. In each panel, the signal deconvolution with five Voigt curves is reported. While the spectrum in (A) was acquired using a 532 nm laser pump to maximize the Raman intensity, the spectrum in (B) was acquired using a 1064 nm laser pump to avoid the intense fluorescence signal due to the presence of the glutaraldehyde Schiff bases in the HSA-MPs.

**Table 1:** Secondary Structure Assignment of Amide I Band Component in Proteins^38^ ^a^

secondary structure	component position range (cm^–1^)	center (cm^–1^)
β-sheet (ν_1_)	1620–1648	1634
α-helix (ν2)	1649–1660	1654
random coil (ν3)	1660–1665	1662
β-sheet (ν4)	1665–1680	1672
β-turn (ν5)	1680–1699	1690

a The variability of the different ν_*i*_ within relatively large spectral ranges is due to the variance of proteins compositions and the relative chemical bonds rearrangement.^39^

To interpret our Raman data, it is worth summarizing that (i) the ν_1_ and ν_4_ components are attributed to β-structures, with ν_1_ being related to parallel and ν_4_ to antiparallel β-sheets, typically involved in structural stability and intermolecular interactions; (ii) the ν_2_ component is associated with α-helix structures, commonly related to ligand binding and conformational flexibility; (iii) the ν_3_ band corresponds to random coils, indicative of disordered or flexible regions; and (iv) the ν_5_ component is linked to β-turn structures, often located in loop regions and involved in protein folding or surface accessibility. The HSA spectrum (Figure 5A) is dominated by the ν_2_ component (∼70% of the total Amide I band) corresponding to α-helices, in agreement with literature findings.^39,40^ In contrast, the Raman spectrum acquired on HSA-MPs (Figure 5B) shows a change in the spectral weights and a moderate shift of the ν_*i*_ components. In particular, the intensity of ν_4_ increases up to 40% of the total Amide I band, at the expense of the intensity of ν_2_, which counts for 50% of the total integrated intensity. In addition, ν_1_ and ν_3_ show a blue-shift of 5 and 7 cm^–1^, respectively, both lying within the spectral range of the assigned secondary structure.

To confirm these results, we performed FTIR spectroscopy on HSA and HSA-MPs diluted in D_2_O heavy water. In fact, FTIR offers the advantage of being unaffected by fluorescence interference, which, when present, could compromise Raman measurements. Additionally, the use of D_2_O helps to eliminate the interference from the water scissoring vibration band, which typically overlaps with the Amide I region. As a result, the Amide I band in the FTIR spectra appears as a well-resolved, collection of individual contributions, preserving the integrity of the spectral information related to the protein’s secondary structure, as reported in Table 2. In Figure 6, we show the FTIR absorption spectra of the Amide I band spectral region for (panel A) HSA, and (B) HSA-MPs. Notably, the two FTIR spectra are related to each other very similarly to how the corresponding Raman spectra are related. Also in the FTIR data one can see that the spectral evolution following the HSA-MPs synthesis is characterized by a 44% increase in the spectral weight of the β-sheet-related components and by the decrease of the α-helix component, that was dominating the HSA FTIR spectrum, as expected.^41–44^ We can thus conclude that both Raman and FTIR results are consistent in showing that the secondary structure of HSA-MPs differs significantly from that of HSA, with a greater proportion of β-sheets and fewer α-helices. We attribute the onset of this structural change to the CCD procedure, and specifically to the cross-link steps during HSA-MP formation.

**Table 2:** FTIR Spectral Intervals and Centers of the Secondary Structures of Proteins in D_2_O^45^

secondary structure	FTIR component position range in D_2_O (cm^–1^)	center (cm^–1^)
β-sheet (ν_1F_)	1615–1638	1630
α-helix (ν_2F_)	1642–1660	1652
random coil (ν_3F_)	1639–1654	1645
β-sheet (ν_4F_)	1672–1694	1679
β-turn (ν_5F_)	1653–1691	1671

**Figure 6:**
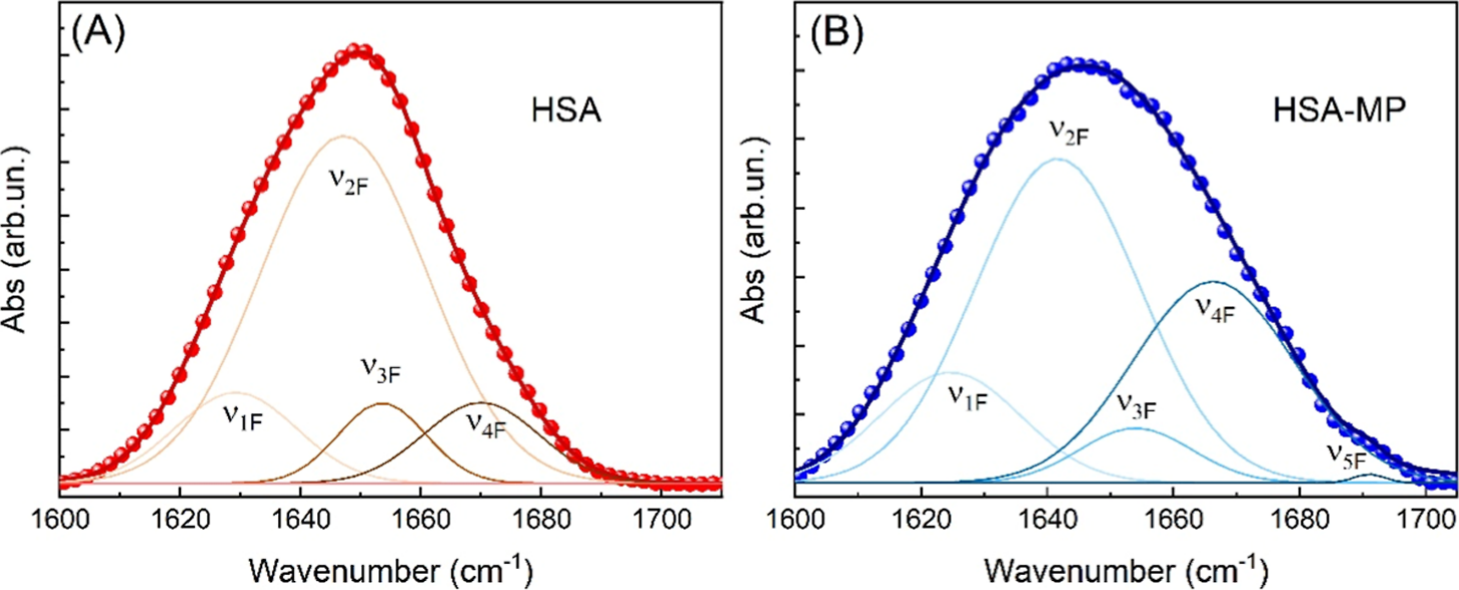
FTIR spectroscopy analysis of HSA (A) and HSA-MPs (B) secondary structure in the Amide I band.

To verify this hypothesis, we have investigated the changes observed in the HSA Raman spectra after precipitation with MnCl_2_ and Na_2_CO_3_ (Figure 7). Indeed, salt-induced precipitation is known to alter protein solubility and folding by modifying electrostatic interactions, hydration layers, and intramolecular hydrogen bonding. Divalent cations such as Mn^2+^ and carbonate anions can promote structural rearrangements in HSA, potentially shifting its secondary structure equilibrium.^41,46^ Consistently with what discussed in Figure 5B, the intensity of the ν_2_ component associated with the presence of α-helices decreased in comparison with the native HSA, attaining a relative abundance of approximately 58%. On the other hand, the spectral weight of the ν_1_ and ν_4_ components, related to β-sheets structures, increased, reaching a relative abundance of ∼32%. These results indicate that the salt-induced precipitation process leads to an increase in the β-sheet content with the loss of the α-helix one, thereby altering the native secondary structure of HSA, similarly to what we observed after the CCD-synthesized HSA-MPs.

**Figure 7:**
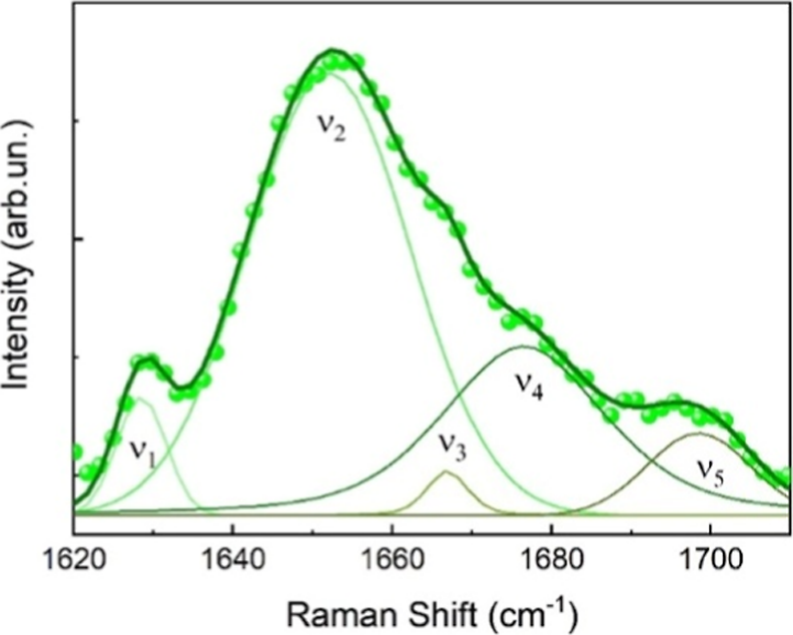
Raman spectrum of the Amid I of HSA after the precipitation with MnCl_2_ and Na_2_CO_3_. Spectral components associated with α-helices (ν_2_) and β-sheets (ν_1_ and ν_4_) structures are reported.

Given the clear evidence of secondary structure alteration following HSA-MPs synthesis, we further investigated how these conformational changes affected the HSA-MP binding capability. This study was conducted to evaluate the functional suitability of the HSA-MP as drug delivery platform, in which the maintenance of correctly folded FA binding sites is essential to ensure efficient drug binding and controlled release. To this end, we compared the binding behavior of HSA and HSA-MP with a prototypical albumin ligand, i.e., hemin, which normally binds to the FA1 site of HSA.^9^ To this end, UV–vis spectroscopy in the 380–500 nm spectral range was used (Figure 8). The spectral absorption of the HSA:hemin complex (orange) featured a characteristic Soret band centered at 404 nm, with an intensity that depends on the amount of hemin bound to the protein.^29,47^ In contrast, the UV–vis spectrum of HSA-MPs incubated with hemin (cyan) showed a shift of the Soret band to 393 nm, a wavelength compatible with the UV–vis spectra of a free hemin.^48,49^ Indeed, the spectrum of free hemin (red) showed a shoulder at 388 nm, while noncomplexed HSA-MPs (blue) displayed a broad, low-intensity signal with no distinct features. These results suggest that, although the HSA-MP can still interact with hemin to some extent, this interaction is nonspecific and lacks the defined coordination observed in HSA. The spectral shift and broadening suggest that the HSA binding sites that are responsible for its strong, specific interaction with hemin are altered or lost during the MP formation.

**Figure 8:**
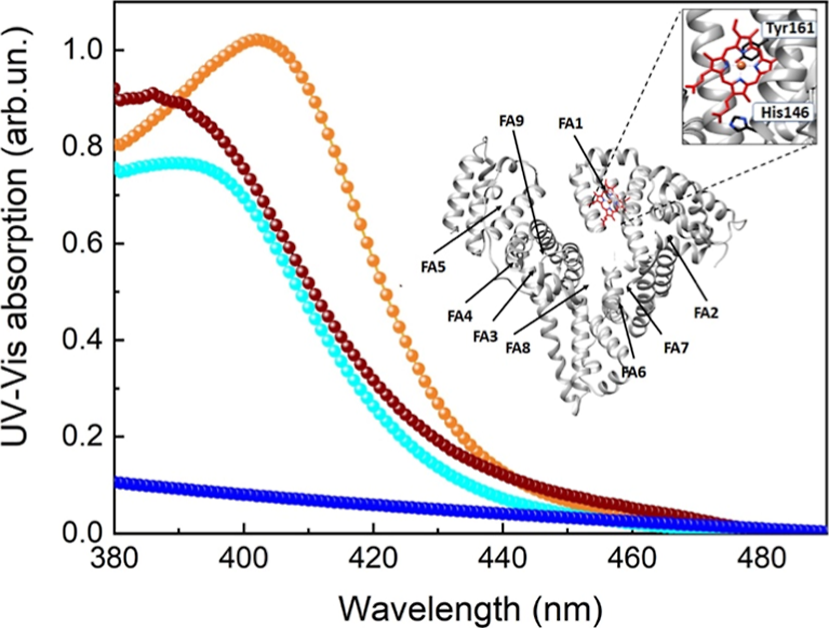
HSA and HSA-MP binding to hemin. UV–vis spectra of free hemin (red data), HSA:hemin complex (orange), HSA-MP:hemin (cyan), and HSA-MP (blue). In the inset, the 3D structure of HSA bound to heme (PDB ID: 1N5U)^50^ is reported. The His141 and Tyr146 residues, coordinating the heme-Fe atom, are shown. The picture has been drawn using the UCSF Chimera package.^51^

Therefore, we conclude that CCD-synthesized HSA-MPs do not retain the same hemin-binding capability as native HSA. Notably, preincubating HSA with hemin prior to MP formation does not overcome the structural instability of HSA during MP formation (data not shown). This result has important implications for HSA-MP use as drug carriers. Indeed, the fact that HSA molecules localized on the surface of MPs cannot bind to plasma ligands (e.g., heme, drugs, metals, bacterial toxins) prevents them from transporting unwanted ligands to cellular targets. Further optimization of the MP synthesis process is necessary if the binding capacity of the HSA molecules present on the MP surface is to be exploited.

## 4 Conclusions

In this study, we conducted a comprehensive structural and functional characterization of the HSA-MPs synthesized using the CCD method. Our results demonstrate that the CCD process consistently yields HSA-MPs with a distinctive peanut-like morphology, submicron size, moderate polydispersity, and a stable negative zeta potential conducive to colloidal stability. The AFM analysis confirmed the reproducibility of this morphology, revealing two distinct size populations with a well-defined volume distribution. Complementing this, QNM investigation showed that the HSA-MP exhibits uniform mechanical properties across the population, with high stiffness, dissipation, and adhesion. These mechanical attributes underscore the robustness of the HSA-MP architecture, highlighting its potential as a stable platform for biomedical applications.

However, our chemical-functional analyses revealed a critical trade-off. Raman and FTIR spectroscopy indicated significant alterations in the secondary structure of HSA upon MP formation, including a marked reduction in α-helical content and a concurrent increase in β-sheet structures. These structural changes, occurring during the precipitation phase of CCD synthesis, appear to compromise the functionality of the protein. Indeed, UV–vis spectroscopic analysis revealed a marked loss of hemin-binding capability in the HSA-MPs, as evidenced by the disappearance of the corresponding Soret band and the emergence of a broader, red-shifted peak. This result appears particularly relevant considering that all the albumin FA sites bind drugs affecting their pharmacokinetic and pharmacodynamics.^10^

Taken together, our findings highlight a key duality: while HSA-MPs produced via CCD possess desirable morphological and mechanical properties, their compromised binding functionality limits their direct use as carriers for drugs that must be loaded on the MP and therefore rely on a correctly folded HSA for their proper delivery. However, it should be remembered that HSA plays an important role in transporting numerous endogenous and exogenous ligands.^1^ Therefore, when considering the potential therapeutic use of HSA-MPs for transporting drugs to target cells, the loss of the correct secondary structure of the HSA molecules on the surface of the MPs prevents them from binding to the ligands present in the plasma, which would then be transported to target cells together with the drug localized in the HSA-MP. In this way, cancer cells, for example, internalize HSA-MP and the drug localized within it.^24^

Finally, the robust structural core of HSA-MPs could serve as a foundation for hybrid systems. For instance, a functional outer layer composed of or engineered HSA could be deposited to restore binding capabilities while retaining the mechanical advantages of the core. Future research should build on these findings to explore and develop such modifications, ultimately unlocking the full potential of HSA-MPs for advanced therapeutic applications.
